# Dr. Alder's Proofs of the Proximate Causes of Diseases, from the Practice of Physic

**Published:** 1809-10

**Authors:** 


### Dr. Alder's *Proofs of the proximate Causes of Diseases, from the Practice of Physic.*

( In Continuation. )

It appears it would be improper for me to dilate much here upon the articles of cheerfulness and good air as preventives of disease, as I mean to notice them rather fully at as remedies for disease, and their opposites as the causes of disease. The article of exercise too, I think, need not be much more fully noticed here. All these are good, so far as they make respiration free and easy, and transmit the blood from the veins into the arteries, and thence regularly and equably all over the body, and they are best conjoined. Exercise, indeed, is seldom of any advantage without one of these, and sometimes not without both; for it hurries the blood up into the lungs, and if the respirating organs, while it does so, are clogged either by reason of very deeply afflicting passions, or a very remarkably impure state of the air, or by both, so that the blood cannot pass through the lungs so fast as it arrives there; exercise, by being long persisted in this case, and the respirating organs not becoming more free under it, *it must do harm.* And I think that, in general, mental affliction and bad air together, will so clog the respirating organs, that exercise cannot do good; though I believe, one of them (unless very severe) for the most part will not. For exercise *in itself,* has naturally a tendency to invigorate the muscles of respiration, and to make *more air respired in a given time,* than there was before it was used.

### *Proofs from the Treatment of Diseases.*

The remedies employed for the cure of fevers, appear somewhat contradictory; they are, bleeding, purging, mercury, the cold bath, wine and bark, opium, electricity, pure air, pleasing the mind, keeping the stomach and bowels in good order, the tourniquet, and some others of less powers. But all of these, I conceive, however contradictory they may appear, may be so used as to do good, and to coincide with my theory.

I apprehend that the statements of Dr. Rush and Dr. Jackson, in favour of bleeding in fever, are faithful, though at the same time I am equally persuaded that wine, opium, and bark, have done good; and that these, as well as several others of the remedies named above, *do appear* contradictory. Every one knows that fevers often demand different or opposite remedies, from being different in their kind; and that fevers are often said to be different in kind, chiefly or solely, because they have yielded to different or apparently opposite remedies. But yet, at the same time, I believe most know, that from the symptoms of these fevers, and the time and place of their occurrence, there often is sufficent ground to say, that the same kind of fever has yielded to *apparently* opposite remedies.

It shall be my task only to shew in what way these different remedies *may be* of service in fevers.

*Bleeding.*—In bleeding, there *often* is an immense difference between venæsection and arteriotomy, and particularly in fevers; for in fevers (and in many other diseases) the veins will often be found turgid, while the arteries are not; and often too when the arteries are ill-supplied, (shewn by the pulse being very small and feeble). In these cases arteriotomy would (at least in general) be absurd. But in these cases venæsection has often been used with success, the respiration becoming more free after it, and the pulse fuller and stronger. Many cases of asthma, of enteritis, and of pneumonia, &c. as well as of fevers, yield confirmation of this truth. And many cases of apoplexy and epilepsy, as well as many more of fever, would do so too, did not the great turgescence of the veins prevent the blood from flowing freely from the arteries into them, *in consequence of which the arteries are full as well as the veins.* That full labouring kind of pulse which, sometimes, is not much different to the touch from a natural pulse, and which nevertheless is attended with a complete debility of the body, and is called, when in its extreme, "*the apoplectic pulse,*" is owing *to the arteries not being able to empty their contents regularly into the veins;* which indeed is often proved by the veins (particularly the jugulars) at this time swelling and violently beating. I maintain then, that venæsection is an operation well calculated to remove disease in many cases, although the arteries are ill-supplied, and the body is feeble: and that in many other cases, when the arteries are full, venæsection is preferable to arteriotomy; their fulness being owing to an impeded outlet for their blood into the arteries.

That venæsection is good in fevers, when the *veins in the head* are so very turgid as to threaten apoplexy, epilepsy, mania, or violent delirium, I believe *most* will admit; for it has so commonly been had recourse to in these cases, and has so usually been found successful in them, that but few can deny it. But yet we find, that much inapplicable or false reasoning has been made to bear against it in these cases; and, what is more strange, by men *who use it in such cases in practice.* The present Dr. Monro, sen. has found, that the brain is not compressed under a heavy weight; and, as it is surrounded on all sides by an almost unyielding bone, many ask, how can the head have more blood at one time than another? But this question is nothing to the purpose; for, if the veins veins are overloaded, so as to cause danger, we must deplete them, although the arteries should have less blood than usual; and if the blood press upon the brain, so as to do much mischief, and threaten much more, we must prevent it from making that præternatural pressure, although we know it cannot compress it; and cannot, of course, be more abundant in the head than it was before. Some too, greatly relying upon this kind of reasoning, and other kinds equally bad, grow bold enough to assert, that the bad symptoms which arise in these cases, are owing to want of energy in the brain, and are for giving this energy by stimuli. But, if the brain have lost part of its energy in such cases, we must restore to it what energy it has lost *by removing the causes which have deprived it, and do deprive it, of that energy*; but we cannot *always* say in such cases, that the brain has lost any energy; for while a pressure upon the brain, either from blood or from any other cause, sometimes produces debility, it sometimes produces the reverse, as is exemplified in many instances of delirium, phrenitis, and mania. Some object to bleeding in cases of fever, where they allow it would *immediately* do good, by saying, it takes that blood away which will *afterwards* be wanted. And Dr. Gregory, after stating this objection, says, he has known several cases of fever where this remedy had been employed, end in dropsy; which termination of them he attributes to the bleeding. But it must be observed, that this objection, is in part a matter of *opinion*, and in part only a matter of fact. If bleeding remove a state of the body, which immediately threatens life, (and the supporters of this latter objection allow that it often immediately does good) it would be prudent in such cases to use it, although the blood taken away would afterwards be wanted, and although the patient, in recovering, might run *some hazard* of dropsy. But I indeed am somewhat inclined to doubt, whether those patients labouring under such a state of fever as can be much and instantly relieved by venæsection, would have more strength when the violent symptoms had abated, if bleeding, (that is, venæsection) was not used, than they would have if it was; (provided that in such cases they got over the violent symptoms:) and, I am somewhat unconvinced, that when dropsy comes on after a fever has subsided, in which bleeding has been successfully used, that the dropsy is to be attributed to the bleeding; for it appears to me, that the supporters of both these ideas are led by what they think theory, much

 more more than they are by plain facts, and their theory seems wrong.

*These Gentlemen seem to think, that debility is a necessary consequence of loss of blood; and that the dropsy is the consequence of debility:* but neither of these positions is quite correct. For, in innumerable cases, it is *disease* which causes weakness; and in these cases strength cannot be restored, *but by removing the disease.* And that which removes the disease, *may be purging, or bleeding, &c.* If great weakness, or dropsy, appear in the latter end of fevers, in which purging or bleeding has been used, this is by no means *proof* that such were occasioned by the bleeding: there seems no other ground for supposing that they were than merely this, that *they followed it*; and things succeeding others are not necessarily to be attributed to them. Great weakness and dropsy, &c. do often follow fevers, whatever may have been their mode of treatment: but I should have much less apprehension of them after a fever *well cured,* than after one which was suffered to go on pretty much in its own way; provided both were equally dangerous, and other circumstances were equal. I do maintain, that when bleeding is properly employed in fevers, it does not cause debility. I might go on too to shew, that the dropsy, which sometimes follows fevers, is not exactly the effect of debility, but that it arises from a turgescence and subsequent relaxation in the excretories and vessels terminating in them; and, that though the debility *may occasion the relaxation,* it was venous plethora which caused the previous turgescence; but debility not being the consequence of a judicious use of bleeding in fevers, renders this unnecessary.

Bleeding may be used with propriety in fever, not only to relieve the head, but also to relieve the lungs, the liver, the intestines, or any part which is becoming dangerously congested. And all these parts frequently become dangerously congested in fevers; but it must not be understood that I am speaking of the common fevers of this country, or of any country; or of any particular kind of fever; *but of fevers in general.* And these perhaps are not in a practical point of view, to be treated on any *one* principle more efficaciously than this one which depends upon the manner in which venous congestions affect these organs.

In those bad fevers in which (as we have seen from dissections) that venous congestion overloads the lungs, the liver, the head, intestines, &c. or some of them, so as quickly to produce produce the most extensive and dreadful inflammations, &c. bleeding (and that very early) often seems the only remedy which can be depended on. But as to any minute description of the cases in which it should or should not, or need not be used; of the time for using it; of the quantity of blood to be drawn at once; and of the number of repetitions, I fear I have not sufficient room for it. And indeed, such are the contradictions among authors and practitioners, and such is the danger of misapplying my principles in such a disquisition, that I do not think I could do it justice, without taking a very extensive as well as minute prospect of it; while at the same time, for the very same reasons, there is but little chance that any thing which I might advance would much assist me in my present object, as this is *not to lay down a plan for practice,* but to ascertain which are good practices; and to shew, according to my system, *why they are so.*

However, I think that as both the trials made, and my Theory, suggest that venæsection may and should be used in the cases specified, so also they will assist a person at the bed-side, to discern these cases, and also to time, modify, and proportion the bleeding to such cases; and when I purposely treat of the practice of physic, (as I mean shortly to do) I shall enlarge on this subject.

I do not think that bleeding is generally requisite in any of the following cases; in mild remittents and in mild continued fevers; in intermissions, or even in remissions; or in the sweating stage. I think too, it is seldom requisite for the mere purpose of checking arterial action. And I could adduce some instances in which it has done injury in several of these cases. Dr. Lind, for example, mentions one case where it was injurious in the remission of a fever, for which fever, (as appears by other cases) it was proper; and I can give no better account of the cause of the disrepute into which venæsection in fevers had fallen among many, than that it was usually contemplated *as used in these and similar improper cases.* It is not required in these cases, because the venous congestion has subsided, is subsiding, or will subside, without any danger; and it is pernicious in some of them, as it might facilitate or aggravate its return. For example, if used in an intermission, it would prevent the heart and arteries from receiving their due supply of blood, and would of consequence sink the pulse and strength below the natural standard, or the state they were in previously; by which means the blood would not be derived sufficiently to the external parts; these would contract, and a new congestion would be much more readily readily formed in the lungs. This happened in Dr. Lind's case. It is seldom useful, and frequently not safe to check the free flow of blood by the arteries; as this takes it out of the veins; and danger seldom arises from mere arterial action. In most cases of fever, (excepting those where there is a very considerable phlegmonic inflammation) the patient is safest when the blood gets freely into the arteries, and in many he is quite safe.

*September* 5, 1809.

[ To be continued. ]

